# Clinicopathological Parameters and Immunohistochemical Profiles in Correlation with MRI Characteristics in Glioblastomas

**DOI:** 10.3390/ijms252313043

**Published:** 2024-12-04

**Authors:** Tamás-Csaba Sipos, Kövecsi Attila, Lóránd Kocsis, Adrian Bălașa, Rareș Chinezu, Beáta Ágota Baróti, Zsuzsánna Pap

**Affiliations:** 1 Department of Anatomy and Embryology, George Emil Palade University of Medicine, Pharmacy, Sciences and Technology of Târgu Mures, 38 Gheorghe Marinescu Str., 540142 Târgu Mures, Romania; tamas.sipos@umfst.ro (T.-C.S.); lorand.kocsis@umfst.ro (L.K.); zsuzsanna.pap@umfst.ro (Z.P.); 2 Doctoral School of Medicine and Pharmacy, George Emil Palade University of Medicine, Pharmacy, Sciences and Technology of Târgu Mures, 540142 Târgu Mures, Romania; 3 Pathology Department, George Emil Palade University of Medicine, Pharmacy, Sciences and Technology of Târgu Mures, 38 Gheorghe Marinescu Str., 540142 Târgu Mures, Romania; 4 Pathology Department, County Emergency Clinical Hospital of Târgu Mureș, 540136 Târgu Mures, Romania; 5 Neurosurgery Department, George Emil Palade University of Medicine, Pharmacy, Sciences and Technology of Târgu Mures, 38 Gheorghe Marinescu Str., 540142 Târgu Mures, Romania; adrian.balasa@umfst.ro (A.B.); rares.chinezu@umfst.ro (R.C.); 6 Neurosurgery Department, County Emergency Clinical Hospital of Târgu Mureș, 540136 Târgu Mures, Romania; 7 Radiology Department, George Emil Palade University of Medicine, Pharmacy, Sciences and Technology of Târgu Mures, 38 Gheorghe Marinescu Str., 540142 Târgu Mures, Romania; beata.baroti@umfst.ro; 8 Radiology Department, County Emergency Clinical Hospital of Târgu Mureș, 540136 Târgu Mures, Romania

**Keywords:** glioblastoma, MMP-9, MRI, *IDH1*, *ATRX*, p53, Ki67, CD34, CD105

## Abstract

Glioblastoma is considered the most aggressive tumor of the central nervous system. The tumor microenvironment includes several components, such as endothelial cells, immune cells, and extracellular matrix components like matrix metalloproteinase-9 (MMP-9), which facilitates the proliferation of endothelial cells with pro-angiogenic roles. The MRI characteristics of glioblastomas can contribute to determining the prognosis. The aim of this study was to analyze the relationship between tumor angiogenesis in glioblastomas in association with MMP-9 immunoexpression. The results were correlated with the Ki-67 proliferation index, p53 immunoexpression, and the mutational status of *IDH1* and *ATRX*, as well as MRI imaging data. This retrospective study included forty-four patients diagnosed with glioblastoma at the Department of Pathology, Târgu Mureș County Emergency Clinical Hospital. MMP-9 immunoexpression was observed in approximately half of the cases, more frequently in patients over 65 years old. Comparing the imaging data with the immunohistochemical results, we observed that the median tumor volume was higher in glioblastomas with *IDH1* and p53 mutations, *ATRX* wild-type status, negative MMP-9 expression, and high Ki-67 proliferation indexes. The median values of MVD-CD34 and MVD-CD105 were higher in cases with extensive peritumoral edema in the contralateral hemisphere. Additionally, *ATRX* mutations were frequently associated with a more pronounced deviation of the median structures. To statistically validate the associations between MRI and the histopathological features of glioblastomas, further studies with larger cohorts are required.

## 1. Introduction

Glioblastoma is the most aggressive type of malignant intracranial tumor of glial origin in adults, characterized by a high rate of local recurrence. The standard treatment for glioblastoma is multimodal; however, the survival rate typically does not exceed 15 months from the time of diagnosis [1,2,3,4,5].

The invasive characteristics of glioblastomas are closely associated with the tumor microenvironment. This microenvironment consists of several components, including tumor glial cells, endothelial cells, immune cells, and extracellular matrix elements [6,7]. Matrix metalloproteinases (MMPs) are zinc-dependent proteolytic enzymes, also referred to as endopeptidases, that are involved in the remodeling and degradation of the extracellular matrix. They degrade components such as collagen, fibronectin, and laminins by utilizing cytokines and growth factors [8,9,10,11].

Matrix metalloproteinase 9 (MMP-9), also known as type IV collagenase, gelatinase, or gelatinase B, plays a key role in the disruption of the blood–brain barrier [12] and facilitates the release of local tumor growth factors associated with the promotion of angiogenesis [13]. Additionally, MMP-9 promotes the proliferation of endothelial cells by releasing vascular endothelial growth factor (VEGF), leading to increased vascular permeability; thus, its pro-angiogenic role in glioblastomas contributes significantly to neovascularization [14]. MMP-9 is also involved in remodeling the tumor microenvironment, which may enhance tumor invasion and progression, and is associated with a poor prognosis [15,16,17].

Magnetic resonance imaging (MRI) is widely used as a non-invasive diagnostic tool for central nervous system tumors. MRI plays an advanced role in the management of patients with glioblastoma (GBM) [18]. Imaging data, alongside the extent of surgical resection, use of adjuvant therapy, and levels of tumor biomarkers, can serve as prognostic factors [19,20]. Anatomical characteristics, such as tumor location and laterality, have been compared with specific molecular alterations in glioblastomas, including VEGF and MGMT [21]. Some studies have shown that glial tumor cells in glioblastomas can also be present in the peritumoral edema area [22].

The development of peritumoral edema is influenced by several factors that act through mechanisms promoting the release of substances that increase vascular permeability, such as vascular endothelial growth factor (VEGF), associated with elevated expression of metalloproteinases, which can further stimulate tumor angiogenesis. Another contributing factor to the onset of peritumoral edema is the overexpression of Aquaporin 4 by tumor glial cells through a mechanism that is not yet fully understood [23,24,25].

Regarding angiogenesis, which can contribute to the development of peritumoral edema, several phenotypic and functional characteristics of endothelial cells in glioblastoma have been demonstrated. These cells form tight connections with tumor cells, altering tissue homeostasis towards a microenvironment with a structure and function more favorable to the tumor. In newly formed vessels within the tumor stroma, most endothelial cells are CD31 and CD34 immunopositive and co-express VEGF. In contrast, most endothelial cells in the peritumoral edema area express CD31 and CD34, but lose VEGF immunoexpression. The decreased expression of VEGF in these endothelial cells may be a response to the therapeutic failure of anti-angiogenic drugs [26].

Imaging data, such as tumor size or location associated with peritumoral edema, have been correlated with the degree of tumor invasion or patient survival. However, studies have shown conflicting results on this topic; some consider peritumoral edema to be a poor prognostic factor, while other authors have reported inconclusive data [27].

The aim of this study was to analyze the relationship between tumor angiogenesis in glioblastomas in association with MMP-9 immunoexpression. The results were correlated with the Ki-67 proliferation index, p53 immunoexpression, and the mutational status of *IDH1* and *ATRX*, as well as MRI imaging data.

## 2. Results

### 2.1. MMP-9 Immunoexpression in Glioblastoma

**Figure 1:**
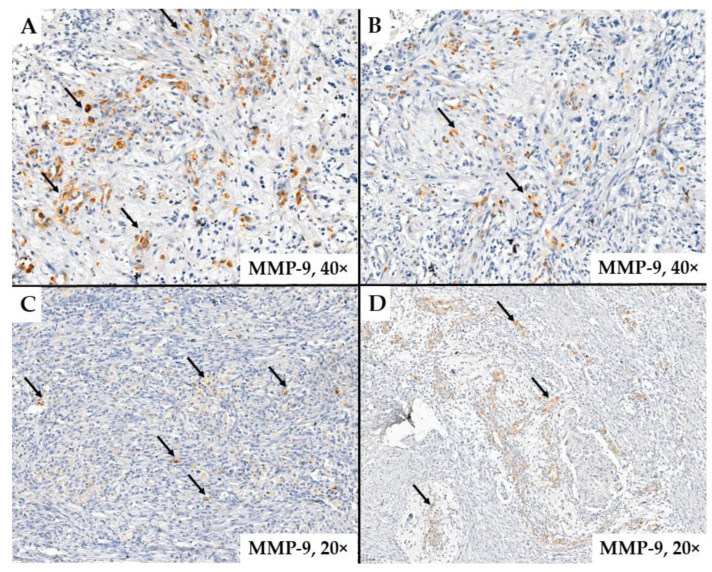
MMP-9 immunoexpression in glial cells is predominantly observed around the tumor stroma adjacent to neoformed vessels (arrows). The images suggest glial structures expressing MMP-9. This expression appears to be variable, as observed in images **A** and **C**. In images **B** and **D**, areas resembling glomerular or vascular garland structures are visible. Within these structures, clusters of glial cells expressing MMP-9 can be identified.

**Figure 2:**
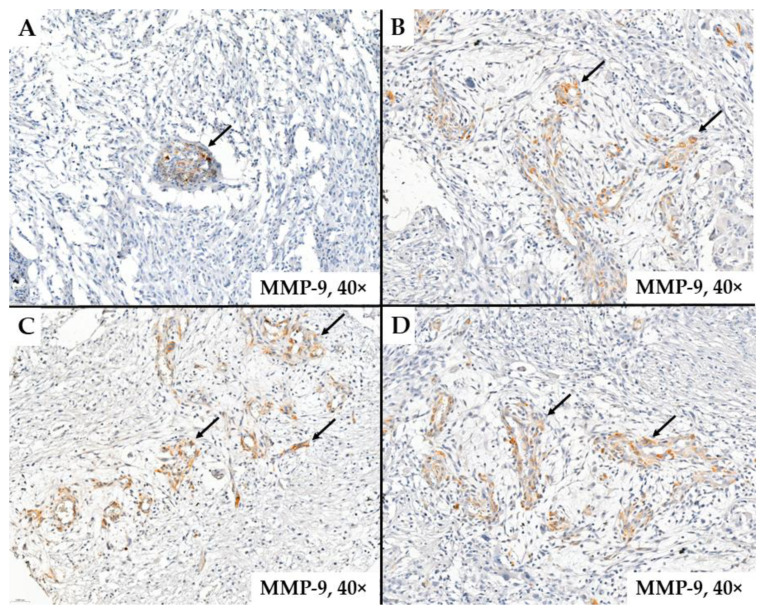
Representative cases of MMP-9 expression. MMP-9 immunoexpression in glial cells is predominantly observed around the tumor stroma adjacent to neoformed vessels (arrows). In the images on the right, small vessels lined by endothelial cells are visible, with some showing positive MMP-9 staining. This suggests the involvement of matrix metalloproteinase in the angiogenesis of the tumor microenvironment in glioblastoma cases. All the images (**A**–**D**) show various shapes or sizes of the newly formed vessels. Glomerular structures can be observed in image **A**, while vascular garland formations are visible in image **C**. Vascular clusters can also be identified in images **B** and **D**.

In our study, we included 44 patients diagnosed with glioblastoma. Over 50% of the glioblastomas examined exhibited MMP-9 immunoexpression. It can be observed that in some glial cells, the cytoplasmic immunoexpression was variable, with differing reaction intensities (Figure 1). We observed endothelial cells with MMP-9 immunoexpression, particularly in areas where glomeruloid structures were present in the tumor stroma (arrows) (Figure 2).

The expression of matrix metalloproteinase 9 (MMP-9) varied widely with patient age. It was observed that as age increased, MMP-9 immunoexpression was more pronounced in the studied glioblastomas. Among patients under 50 years of age, MMP-9 immunoexpression was present in 37.5% (6/16) of cases, whereas 62.5% (10/16) of cases showed no MMP-9 immunoexpression. In contrast, among patients over 65 years of age, there was a notable increase in MMP-9 immunoexpression, with 78.57% (11/14) of cases showing positive staining and only 21.42% (3/14) of cases showing no MMP-9 immunoexpression. The number of cases with or without MMP-9 immunoexpression was equal among patients aged 50 to 65 years. As age increased linearly, no statistically significant correlation was found between MMP-9 immunoexpression and patient age (*p* = 0.28, r = 0.16). Regarding gender differences, a predominance of MMP-9 immunoexpression was observed in female patients, at 59.09% (13/22). No statistically significant correlation was found between MMP-9 immunoexpression and either the age group or gender of the patients (*p* = 0.07, *p* = 0.54) (Table 1).

Regarding localization, almost 45.83% of the cases with MMP-9 immunoexpression were found in the temporal lobe (11/24). In the occipital lobe, MMP-9 immunoexpression was observed twice as frequently as cases without MMP-9 immunoexpression. MMP-9-positive cases were more common in the right cerebral hemisphere, at a rate of 58.33% (14/24). Conversely, MMP-9-negative tumors were more frequent in the left hemisphere (12/20, 60%). No statistically significant correlation was found between tumor localization, laterality, and MMP-9 immunoexpression (*p* = 0.91, *p* = 0.22) (Table 1).

The majority of IDH-1 wild-type glioblastomas (57.5%, 23/40) exhibited MMP-9 immunoexpression, in contrast to *IDH1* mutant glioblastomas, where MMP-9 immunoexpression was significantly lower (1/4). Regarding *ATRX* immunoexpression, most *ATRX* wild-type cases also presented MMP-9 immunoexpression, at a rate of 55.88% (19/34). The loss of *ATRX* immunoexpression did not affect MMP-9 immunoexpression, with an equal number of cases showing either presence or absence of MMP-9. No statistically significant association was observed between the presence or absence of *IDH1* or *ATRX* mutations and MMP-9 immunoexpression (*p* = 0.21, *p* = 0.74) (Table 1).

In glioblastomas with mutant p53, MMP-9 immunoexpression was absent in most cases, accounting for 63.63% (7/11). In contrast, MMP-9 immunoexpression was more frequently observed in p53 wild-type glioblastomas (60.60%, 20/33). Regarding the Ki-67 index, it was noted that as the Ki-67 index increased, MMP-9-positive GBM became less frequent, indicating a decline in MMP-9 immunoexpression. GBMs with Ki-67 indexes below 5% were more often associated with MMP-9 immunoexpression, at a rate of 73.33% (11/15), with a ratio of MMP-9-positive to -negative cases of 2.75 (11:4). Conversely, in glioblastomas with Ki-67 indexes above 20%, the proportion of MMP-9-positive cases decreased to 41.17% (7/17), and MMP-9-negative GBMs were more common (10/17, 58.82%). No statistically significant association was found between the Ki-67 index or p53 status and MMP-9 immunoexpression (*p* = 0.16, *p* = 0.17) (Table 1).

**Figure 3:**
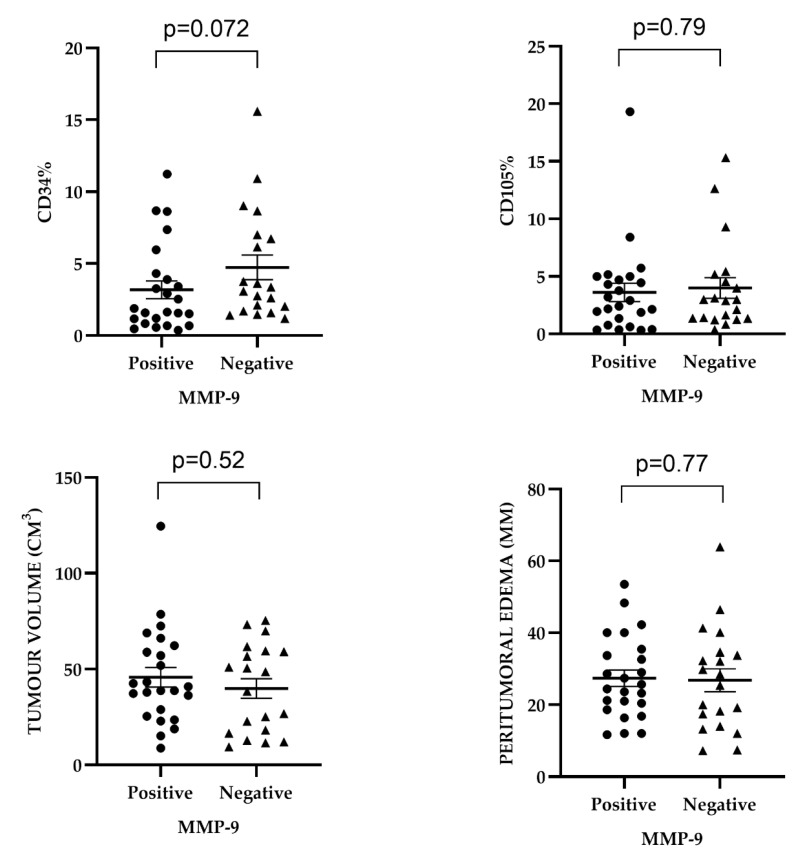
Distribution of MMP-9 in relation to MVD-CD34 and MVD-CD105, peritumoral edema, and tumor volume in glioblastomas.

Considering the microvascular density (MVD) determined by CD34 immunoexpression, we observed that MMP-9 expression was more frequently low or absent in GBM cases with higher MVD-CD34. The median microvascular density measured by CD34 was 1.75% (0.9–4.1) in GBM with MMP-9 expression. In cases without MMP-9 expression, the median microvascular density was higher, at 3.21% (1.7–6.9). Regarding MVD-CD105, cases with MMP-9 expression showed a median microvascular density of 2.65% (0.9–4.9), while those without MMP-9 expression had a slightly higher density of 2.93% (1.3–5.0). No statistically significant association was found between microvascular density measured by CD34 or CD105 and MMP-9 expression (*p* = 0.072, *p* = 0.79) (Table 1). Additionally, a nearly similar distribution of percentage values of microvascular densities, both by MVD-CD34 and MVD-CD105, was observed in MMP-9-positive and -negative tumors (Figure 3).

### 2.2. MRI Characteristics of Glioblastomas

#### 2.2.1. Tumor Size and Tumor Volume

In more than 65% of the studied cases, the maximum tumor diameter was below 5 cm (29/S44), with a median diameter of 4.63 cm. Tumor volume measurements ranged from 8.75 cm^3^ to 124.56 cm^3^, with an average tumor volume of 43.17 cm^3^. 

Regarding tumor size, most glioblastomas with a maximum diameter of less than 5 cm were found in patients over 50 years old (20/44) (*p* = 0.43). In terms of gender, glioblastomas smaller than 5 cm were more common in male patients (18/22). Additionally, the proportion of tumors larger than 5 cm was higher among women (50%, 11/22) compared to men (18.18%, 4/22). A statistically significant association was observed between tumor size and patient gender (*p* = 0.02) (Table 1).

The median tumor volume decreased with increasing age, with the highest tumor volumes recorded in patients under 50 years old. The median tumor volume was higher in women (42.1 cm^3^) compared to men (38.6 cm^3^). However, no statistically significant association was found between median tumor volume and patient age or gender (*p* = 0.859, *p* = 0.477) (Table 2).

The median tumor volume was highest in GBM located in the frontal lobe, followed by the occipital lobe, with the smallest values recorded in the temporal and parietal lobes, which had similar proportions. The median tumor volume was larger in the left hemisphere, with a value of 45.6 cm^3^. No statistically significant correlation was observed between tumor size, median tumor volume, localization, and laterality (*p* = 0.95, *p* = 0.75, *p* = 0.39, *p* = 0.77) (Table 1 and Table 2).

In 65% (26/40) of the cases, we observed *IDH1* wild-type glioblastomas with sizes under 5 cm, while 25% (1/4) of the cases were *IDH1* mutant glioblastomas larger than 5 cm. The median tumor volume was higher in *IDH1* mutant glioblastomas (49.8 cm^3^). Nearly 61.76% (21/34) of *ATRX* wild-type cases had sizes below 5 cm. The ratio of *ATRX* mutant cases with maximum diameters <5 cm versus >5 cm was 8:2 = 4, while in *ATRX* wild-type GBM cases, the ratio was 21:13 = 1.6. The median tumor volume was approximately equal in both *ATRX* wild-type and mutant cases. No statistically significant association was found between tumor size, median tumor volume, and *IDH1* or *ATRX* mutations (*p* = 0.68, *p* = 0.28, *p* = 0.27, *p* = 0.85) (Table 1 and Table 2).

Regarding the p53 mutation, we observed that 66.66% (22/33) of the p53 wild-type cases had tumors smaller than 5 cm, while 36.36% (4/11) of the cases showed the p53 mutation associated with tumor sizes greater than 5 cm. The median tumor volume was higher in p53 mutant glioblastomas (48.6 cm^3^) compared to p53 wild-type cases. A Ki67 index greater than 5% was more frequently observed in tumors smaller than 5 cm (21/29). The median tumor volume was larger in glioblastomas with a Ki67 index above 20%. Concerning the relationship with MMP-9, cases without MMP-9 expression had relatively higher median tumor volumes (43.5 cm^3^ vs. 39.8 cm^3^) compared to glioblastomas with MMP-9 expression. No statistically significant correlation was found between tumor size; median tumor volume; and Ki67 index, p53 mutation, or MMP-9 expression (Table 1 and Table 2, Figure 3).

Regarding microvascular density, no statistically significant correlation was observed between tumor volume and microvascular density measured by either CD34 or CD105. However, the correlation coefficient suggests a positive correlation for MVD-CD34 and a negative correlation for MVD-CD105 (*p* = 0.687, *p* = 0.658) (Table 3).

#### 2.2.2. Peritumoral Edema

Peritumoral edema ranged in size from 7.2 mm to 63.9 mm. The median thickness of peritumoral edema was higher among women (29.1 mm) and in patients aged between 51 and 65 years (30.6 mm). Median values recorded in the temporal lobe were nearly similar to those in the parietal lobe. The right cerebral hemisphere exhibited a higher median value of peritumoral edema compared to the contralateral hemisphere. No statistically significant association was observed between the median values of peritumoral edema and age, sex, tumor localization, or laterality (Table 1 and Table 2).

Differences in the median values of peritumoral edema based on the presence or absence of the *IDH1* mutation were not statistically significant. The median values of peritumoral edema were higher in *ATRX* mutant cases compared to *ATRX* wild-type glioblastomas. The median value of peritumoral edema was higher in p53 wild-type glioblastomas and in cases with Ki67 indexes below 5%. Differences in the median value of peritumoral edema concerning the presence or absence of MMP-9 immunoexpression were not significant. No statistically significant association was found between median values of peritumoral edema and *ATRX*, p53 mutations, or Ki67 index (Table 1 and Table 2, Figure 3).

Regarding microvascular density, which was measured by both CD34 and CD105, no statistically significant correlation was observed between peritumoral edema thickness and microvascular density (*p* = 0.107, *p* = 0.563) (Table 3).

Regarding the margin of peritumoral edema, most cases exhibited rounded margins (27/44). Predominantly in the temporal lobe, we observed edema with irregular contours. Irregular margins were more frequently identified in the right hemisphere, whereas the left hemisphere showed more cases of edema with rounded margins. A statistically significant association was observed between laterality and edema shape (*p* = 0.03) (Table 2).

Most glioblastomas with *IDH1* wild type, p53 wild type, and *ATRX* wild type exhibited rounded edema margins. The ratio of cases with regular versus irregular edema contours was 2 for GBM with Ki67 index below 20% and 1.12 for those with Ki67 indexes above 20%. MMP-9-positive cases more frequently presented irregular margins (10/24) compared to MMP-9-negative cases (7/20). No statistically significant association was found between age, gender, localization, *IDH1*, *ATRX*, p53 mutations, Ki67 index, and MMP-9 with the characteristics of peritumoral edema shape (Table 2).

**Figure 4:**
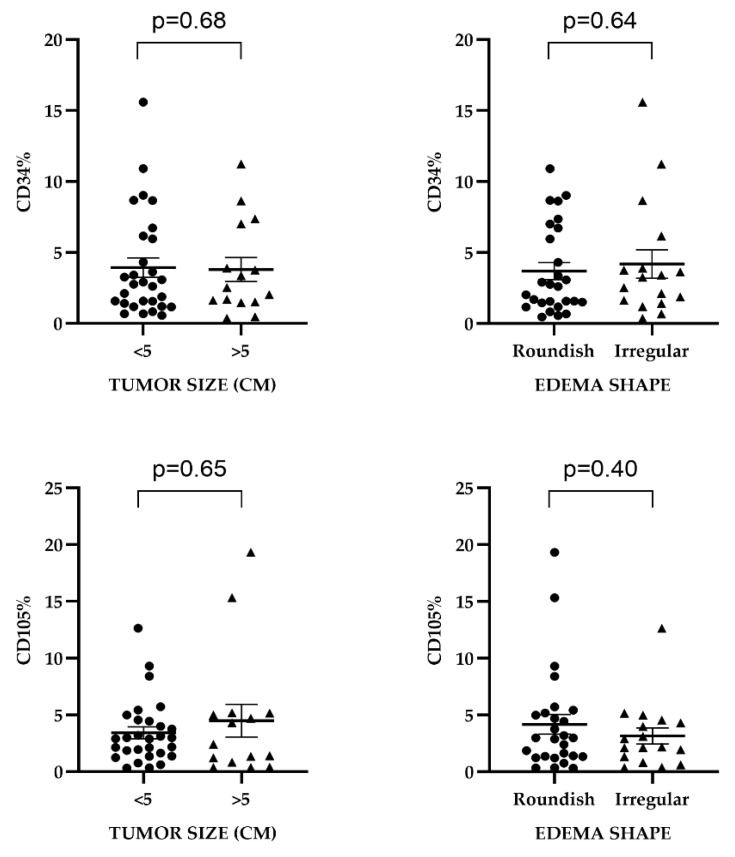
Distribution of microvascular density values in relation with tumor volume and edema shape in glioblastomas.

The median MVD-CD34 value was higher in GBM with irregular peritumoral edema, whereas MVD-CD105 showed higher median values in GBM associated with regular edema contours. No statistically significant association was observed between peritumoral edema margin characteristics and MVD-CD34 or MVD-CD105 (Table 3, Figure 4).

#### 2.2.3. Midline Deviation

The median value of midline deviation was 6.1 mm. In 15.9% (7/44) of cases, no midline deviation was observed, while in 27.27% (12/44) of cases, the deviation exceeded 10 mm. Midline deviation was greater in males and in patients aged between 50 and 65 years. Higher values were noted in tumors located in the frontal lobe, followed by the parietal lobe. Tumors located in the right cerebral hemisphere were associated with more pronounced midline deviation. A statistically significant association was observed between age groups and midline deviation (*p* = 0.006) (Table 2).

*IDH1* mutant glioblastomas, p53 mutant glioblastomas, and *ATRX* mutant glioblastomas exhibited more pronounced midline deviation. Cases with Ki67 indexes above 20% and MMP-9-negative tumors resulted in more severe midline deviation. A statistically significant association was observed between *ATRX* mutation and midline deviation (*p* = 0.035) (Table 2).

There was no statistically significant correlation between midline deviation and the median values of MVD-CD34 or MVD-CD105. However, a negative correlation was noted between the degree of deviation and MVD-CD105, and a positive correlation with MVD-CD34 (Table 3).

Based on the statistical results, this study did not demonstrate a correlation between tumor location and tumor volume, peritumoral edema size, or respective midline deviation (Table 2). No statistically significant correlation was found between tumor volume and peritumoral edema or tumor volume and midline deviation. However, a positive correlation was observed between the peritumoral edema and the midline deviation (Table 4).

## 3. Discussion

Glioblastoma (GBM) is the most common primary malignant brain tumor [28], typically affecting patients over 60 years of age, and is more frequently diagnosed in men. Locationally, the frontal lobe is the most commonly affected, followed by the temporal lobe [29]. Pathologically, glioblastomas are characterized by increased mitotic activity, aggressive invasive behavior, central necrosis, and pronounced angiogenesis. According to the WHO classification, they are classified as grade IV gliomas [30]. Additionally, radiological parameters obtained through preoperative magnetic resonance imaging (MRI) have been shown to have prognostic value, including necrosis volume and peritumoral edema volume [31].

Glioblastomas are lesions that exhibit heterogeneous signal on T1- or T2-weighted MRI sequences, irregular margins, and central areas of necrosis and/or hemorrhage, along with peritumoral edema. The imaging characteristics of a lesion with irregular infiltrative margins, heterogeneous signal, and peritumoral edema are particularly suggestive of a high-grade diffuse glioma [32]. Recurrences are quite common in glioblastomas, and they are associated with detectable peritumoral edema on T2-FLAIR MRI sequences [33]. The edema appears to be a response to angiogenic factors released in the tumor microenvironment, which increase vascular permeability. As tumor proliferation exceeds the native blood supply, resulting ischemia stimulates additional release of angiogenic factors that promote vascular proliferation [34].

In the current study, MMP-9 expression was present in 54.5% of the glioblastomas examined. While we could not demonstrate a statistically significant association between MMP-9 expression and the clinical–pathological parameters studied in glioblastomas, it is noteworthy that MMP-9 expression was more frequently observed in elderly patients; in glioblastomas located in the right hemisphere; in glioblastomas that were *IDH1* wild type, *ATRX* wild type, and p53 wild type; as well as in tumors with lower MVD-CD34 and MVD-CD105.

In terms of the imaging characteristics of GBM, the peritumoral characteristic was associated with irregular margins in approximately 39% of cases. Significant associations were found between more pronounced midline deviation and factors such as age and the presence of *ATRX* mutation. Irregular margins of peritumoral edema were significantly associated with tumor laterality. The presence of edema in the contralateral hemisphere was significantly correlated with increased MVD-CD105 values.

In the study conducted by Roux et al., the average tumor volume was 37.1 cm^3^, whereas in our study, it was 43.17 cm^3^. In total, 63% of the glioblastomas studied by Roux et al. were *IDH1* wild type, compared to over 90% in our study [35]. In contrast, the study by Yu et al. reported a lower median tumor volume of 32.4 cm^3^ [36].

In the study by Wu et al., peritumoral edema with irregular margins was found in 66.7% of GBM cases, whereas in our study, this proportion was only 38.63%. Tumor size exceeding 50 mm was observed in 57.5% of their cases, while in our study, this was only 34%. They described a statistically significant correlation between the degree of peritumoral edema and the form of the edema, in contrast with our study. No significant correlation was found between the degree of edema and sex, patient age, or tumor size, which is similar to our findings. However, in our study, we observed a significant association between tumor laterality and the shape of edema. Major edema and necrosis on MRI are significant prognostic indicators of shorter overall survival (OS) [37].

The increase in matrix metalloproteinase levels promotes the progression of brain tumors, especially glioblastomas. The tumor microenvironment plays a critical role in shaping the prognosis of glioblastoma, with matrix metalloproteinase 9 (MMP-9) serving as a key regulator within this microenvironment [38]. Clinical and experimental studies have demonstrated a correlation between elevated MMP levels and the invasive nature of brain tumors [39,40]. The results of the study by Xue et al. showed a significant enhancement in cellular proliferation in glioblastomas with MMP-9 overexpression [41].

Li et al. analyzed the immunoexpression of MMP-9 in gliomas of various grades, finding that MMP-9 expression was correlated with tumor grade. MMP-9 expression was significantly higher in grade IV gliomas (glioblastomas) compared to grade II and III gliomas. Additionally, patients with glioblastomas showing low MMP-9 expression were generally younger and had higher rates of MGMT promoter methylation and *IDH1* mutations compared to patients with glioblastomas with high MMP-9 expression. Patients with low MMP-9 expression had a longer overall survival (OS) compared to those with high MMP-9 expression [42].

In the study conducted by Zhang et al., gliomas (including 32 glioblastomas) were characterized by frequently having a tumor diameter greater than 30 mm. They found that high levels of MMP-2 in gliomas were associated with a poorer prognosis. Regarding tumor size and grade, positive MMP-2 expression was closely associated with MRI signal uniformity. Severe peritumoral edema was present in more than half of the cases, similar to our study. They observed a statistically significant association between MMP-2 expression in gliomas and tumor diameter as well as peritumoral edema, but not with sex, age, or tumor grade [43].

The Ki-67 antigen directly reflects the degree of cellular proliferation and is closely associated with tumor progression. High Ki-67 index values are linked to a higher grade of malignancy and poorer prognosis. Lower Ki-67 proliferation index values are correlated with the presence of mutations in the isocitrate dehydrogenase gene. Thus, Ki-67 can be considered a prognostic indicator in glioblastomas. Similarly, the p53 protein is also a marker of poor prognosis; however, Ki-67 has a better correlation with radiomic characteristics of GBM on T2-weighted imaging compared to the p53 protein. Additionally, the Ki-67 marker has shown better predictive value in peritumoral areas [44].

The importance of advanced imaging in studying tumor biology through various imaging sequences, such as rCBV (relative cerebral blood volume) and rOEF (relative oxygen extraction fraction), was explored by Wiestler et al. They found that the maximum relative oxygen extraction fraction (rOEF) was higher in glioblastomas compared to grade 2/3 gliomas according to the WHO classification. The expression of HIF1α, which correlates with rOEF, may reflect the level of hypoxia in tumor tissue and stimulate more pronounced local angiogenesis, a characteristic feature of glioblastomas [45].

Another study demonstrated significant correlations between rCBV (relative cerebral blood volume) and microvascular density in IDH-wildtype glioblastomas (GBM) (*p* < 0.001), with rCBV values being 2–2.5 times higher in IDH-wildtype GBM compared to IDH-mutant glioblastomas [46]. Liu et al. found that elevated serum levels of MMP-2 and MMP-9 are correlated with recurrence and show a statistically significant correlation with normal cerebral blood volume (nCBV) and normal cerebral blood flow (nCBF) [47].

Radiogenomics studies the association between molecular phenotypes and specific imaging characteristics to indicate how certain genomic variations might influence the imaging traits of tumors [48]. In the future, non-invasive imaging assessments prior to biopsy or surgery could be an ideal approach for detecting genomic alterations with prognostic significance in GBM. These imaging techniques could also be useful in guiding targeted biopsies [49].

## 4. Materials and Methods

### 4.1. Clinical Data

This retrospective study included 44 patients diagnosed with glioblastoma at the Department of Pathology, County Emergency Clinical Hospital of Târgu Mureș, between 2014 and 2017. The inclusion criteria were as follows: (1) histopathological confirmation of glioblastoma, without any prior diagnosis or oncological treatment for any type of brain tumor; (2) no history of brain biopsy; (3) availability of tumor tissue in at least two paraffin blocks for the determination of immunoexpression of IDH1-R132H, ATRX, CD34, CD105, MMP-9, Ki67, and p53. The histopathological diagnoses were re-evaluated by a neuropathologist according to the 2016 World Health Organization (WHO) classification of central nervous system tumors. Finally, (4) access to preoperative imaging data obtained through MRI (T1-weighted and contrast-enhanced T1, T2, and T2 FLAIR images) was required.

### 4.2. Immunohistochemistry

Surgical specimens were fixed in formalin, embedded in paraffin, and sectioned at a thickness of 3 μm. The obtained sections underwent standard deparaffinization and rehydration procedures. Endogenous peroxidase activity was blocked using a 10 min treatment with 3% H_2_O_2_. Antigen retrieval was performed by pressure steam boiling for 25 min in a citrate solution (pH 6). The following antibodies were used: mouse monoclonal antibody IDH1R132H, clone IHC132 (BioSB, Santa Barbara, CA, USA), dilution 1:25, incubation 60 min; mouse monoclonal antibody ATRX, clone BSB-108 (BioSB), dilution 1:50, incubation 60 min; rabbit monoclonal antibody MMP-9; rabbit monoclonal antibody CD34, clone EP88 (BioSB), dilution 1:100, incubation 60 min; rabbit monoclonal antibody CD105, clone EP274 (BioSB), dilution 1:200, incubation 60 min; mouse monoclonal antibody Ki67, clone MM1 (Novocastra, Leica Biosystems, Deer Park, IL, USA), dilution 1:150, incubation 60 min; mouse monoclonal antibody p53, clone DO7 (BioSB), dilution 1:800, incubation 60 min. The EnVision Flex/horseradish peroxidase (HRP) secondary system (Agilent - Dako, Santa Clara, CA, USA, 30 min) was used for signal amplification, and 3,3′-diaminobenzidine (DAB) was used as the chromogen for primary antibody detection. The slides were subsequently stained with hematoxylin.

### 4.3. Slide Evaluation

The interpretation of immunohistochemical results was supervised by a neuropathologist. Preliminary examination of the slides was performed using an Olympus BX46 microscope, and the slides were subsequently scanned with a 3DHistech PANORAMIC 1000 scanner (Budapest, Hungary). Cytoplasmic immunoexpression of MMP-9, ranging from yellow-brown to dark brown, was recorded as a positive reaction. For the semi-quantitative evaluation of MMP-9 immunoexpression, we considered: (i) staining intensity [0 points (no staining), 1 point (light staining—light brown), 2 points (moderate staining), and 3 points (marked staining—dark brown)] and (ii) the percentage of stained cells [0 points (no stained cells), 1 point (stained cells <25%), 2 points (stained cells 25–50%), and 3 points (stained cells >50%)]. The total score (0–6 points) was calculated by summing these values. A score of 0–2 points indicated the absence of immunoexpression, while a score of 3–6 points indicated a positive immunoreaction [43].

Immunoexpression of p53 and Ki67 was individually evaluated; the Ki-67 proliferation index was determined as the percentage of stained tumor cells (regardless of intensity) out of 1000 cells. The presence of p53 was assessed using the percentage of immunolabeled cells out of 200 cells across 5 fields. p53 was considered negative (wild type) if the immunostaining was <10% and positive (mutant type) if it was >10% of the examined cells [50,51].

Microvessel density was determined based on the immunoexpression of CD34 and CD105. In the tumor tissue, four areas with the highest microvessel density were selected, initially with a low-power objective (×40) and subsequently with a high-power objective (×400). For objective quantification of microvascular density in the tumor stroma, the Slideview software (SlideViewer 2.6.0.166179 software together with QuantCenter 2.3.0.143967—by 3DHistech) was used, and the median values of the four analyzed areas were calculated. The immunohistochemical reaction was considered positive if solitary or clustered endothelial cells, whether participating in lumen formation or not, showed a positive reaction [50].

The expression of the *IDH1* mutation was determined by evaluating tumor cells that were cytoplasmically stained positive, regardless of staining intensity. Cases where ≥10% of the cells were stained were defined as positive (*IDH1* mutant), while cases where this value did not exceed 10% of tumor cells were considered negative (*IDH1* wild type).

In tumor cells, *ATRX* gene mutations result in the loss of nuclear *ATRX* immunoexpression (*ATRX* loss—*ATRX* mutant type), whereas *ATRX* immunoexpression remains preserved in *ATRX* wild type tumor cells, with endothelial cells serving as the endogenous positive control [50,51].

### 4.4. MRI Sequence Evaluation

**Figure 5:**
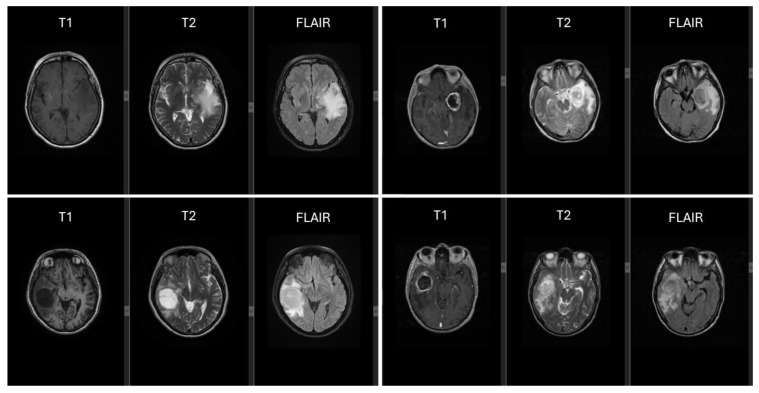
MRI sequences, including T1, T2, and FLAIR, highlight a variety of tumor volumes, locations, and lateralities, each exhibiting peritumoral edema of varying shapes and sizes.

All patients included in this study underwent a standardized preoperative brain MRI using an OptimaTM MR450w GEM 1.5T scanner (GE Medical System, Waukesha, WI, USA). T2-weighted (T2W), T2-FLAIR, T1W, and T1W-CE images were obtained at the Radiology Department of the Clinical County Emergency Hospital in Târgu Mureș (Figure 5).

Using MRI image processing, the tumor volume was determined by manually selecting the region of interest (ROI) with a semi-automatic segmentation method using 3DSlicer 5.6.2 software (https://www.slicer.org/). We also measured the thickness of the peritumoral edema, defined the characteristics of the peritumoral edema margins, assessed the presence of edema in the contralateral hemisphere, and determined the midline shift, similar to protocols described by Wu et al., Palpan et al., and Long et al. [37,52,53].

### 4.5. Statistical Analysis

Descriptive and inferential statistics were performed. The normality of the distribution of continuous variables was tested using the Shapiro–Wilk test. Continuous variables were expressed as medians (25th percentile, 75th percentile), and medians were compared using the Mann–Whitney test. Categorical variables were presented as frequencies, and between-group comparisons were performed using the Chi-square test. A value of *p* < 0.05 was considered significant. Statistical analyses were conducted using IBM SPSS Statistics 22 software (IBM Corporation, New York, NY, USA).

### 4.6. Limitations of the Study

The primary limitation of our study is the small sample size. The analyzed cohort did not provide postoperative or treatment information, so the overall survival rate or the number of recurrences cannot be assessed. In the future, we will likely expand this study to include other MMPs (such as MMP-2) and their serum levels, and the study will have a larger number of participants. We considered IDH1-R132H to be the most relevant mutant variant within codon R132, while R132S, R132C, R132G, and R132L were less common.

### 4.7. Ethics Committee

This study was approved by the Ethics Committee of the Clinical County Emergency Hospital Târgu Mureș under reference number 24494/16 October 2020.

## 5. Conclusions

The immunoexpression of MMP-9, which plays a role in remodeling the tumor microenvironment, was present in approximately half of the studied glioblastomas. It is noteworthy that MMP-9 expression was more frequently observed in elderly patients; in glioblastomas located in the right hemisphere; in glioblastomas that were *IDH1* wild type, *ATRX* wild type, and p53 wild type; as well as in tumors with lower MVD-CD34 and MVD-CD105. 

Comparing the imaging data with the immunohistochemical results, we observed that the median values of MVD-CD34 and MVD-CD105 were higher in cases with extensive peritumoral edema in the contralateral hemisphere. Additionally, *ATRX* mutations were frequently associated with a more pronounced deviation of the median line.

Further studies with larger sample sizes are needed to statistically validate these associations between the imaging and histopathological characteristics of glioblastomas.

## Figures and Tables

**Table 1:** Correlation of clinical and immunohistochemical analyses with MMP-9 immunexpression and tumor size.

N (nr)	Tumor Size			MMP-9		
	<5 cm	>5 cm	*p*	Positive	Negative	*p*
Age						
<50 years	9	7	0.43	6	10	0.07
50–65 years	11	3		7	7	
>65 years	9	5		11	3	
Gender						
Male	18	4	0.02	11	11	0.54
Female	11	11		13	9	
Localization (lobe)						
Frontal	6	4	0.95	5	5	0.91
Temporal	14	6		11	9	
Parietal	5	3		4	4	
Occipital	4	2		4	2	
Laterality						
Right hemisphere	15	7	0.75	14	8	0.22
Left hemisphere	14	8		10	12	
*IDH1* Mutation						
*IDH1* Wild Type	26	14	0.68	23	17	0.21
*IDH1* Mutant Type	3	1		1	3	
p53						
Mutant type	7	4	0.85	4	7	0.16
Wild type	22	11		20	13	
Ki67						
<5%	8	7	0.08	11	4	0.17
5–20%	11	1		6	6	
>20%	10	7		7	10	
*ATRX*						
Mutant type	8	2	0.28	5	5	0.74
Wild type	21	13		19	15	
CD34				1.75 (0.9–4.1)	3.21 (1.7–6.9)	0.072
CD105				2.65 (0.9–4.9)	2.93 (1.3–5.0)	0.79

**Table 2:** Relationship between tumor volume, peritumoral edema, edema shape, and midline deviation based on general and immunohistochemical data.

Data	Tumor Volume (cm^3^)	*p*	Peritumoral Edema (mm)	*p*	Edema Shape (Roundish/ Irregular)	*p*	Midline Deviation (mm)	*p*
Gender								
Male	38.6 (21.8, 57.5)	0.477	20.8 (16.8, 34.6)	0.213	14/8	0.757	7.5 (4.0, 11.5)	0.112
Female	42.1 (24.6, 63.4)		29.1 (21.0, 33.8)		13/9		5.5 (1.5, 8.4)	
Age								
<50	46.9 (28.4, 58.3)	0.859	20.2 (17.8, 31.2)	0.205	14/4	0.336	7.0 (3.7, 11.2)	0.006
51–65	38.3 (26.3, 53.8)		30.6 (27.4, 34.6)		7/7		8.3 (6.3, 10.4)	
>65	33.0 (17.4, 67.6)		24.0 (12.0, 35.4)		8/6		3.0 (1.5, 5.0)	
Localization								
Frontal	57.9 (39.1, 67.6)	0.398	20.7 (16.4, 40.1)	0.434	7/3	0.207	10.8 (3.0, 14.6)	0.381
Temporal	37.6 (18.0, 61.1)		28.7 (19.3, 33.7)		9/11		6.1 (4.0, 9.8)	
Parietal	37.5 (19.8, 50.9)		27.8 (21.2, 40.6)		6/2		6.4 (3.3, 8.7)	
Occipital	40.6 (24.1, 73.6)		19.0 (14.0, 25.7)		5/1		4.5 (1.0, 6.2)	
Lateality								
Right Hemipshere	38.7 (24.6, 61.8)	0.771	28.2 (20.4, 35.4)	0.149	10/12	0.030	8.3 (4.0, 11.5)	0.072
Left Hemisphere	45.6 (18.7, 59.1)		22.4 (14.0, 32.6)		17/5		5.3 (1.9, 8.4)	
*IDH1*								
*IDH1* wild type	38.5 (22.8, 59.3)	0.273	25.5 (18.0, 34.2)	0.775	25/15	0.634	6.1 (3.3, 10.2)	0.539
*IDH1* mutant type	49.8 (42.8, 67.4)		23.6 (15.1, 35.1)		2/2		7.5 (4.3, 12.0)	
*ATRX*								
Wild type	40.6 (18.7, 60.0)	0.857	24.0 (17.4, 33.7)	0.287	21/13	0.599	5.15 (2.0, 9.2)	0.035
Mutant type	39.6 (26.4, 54.8)		30.8 (18.2, 41.3)		6/4		8.8 (5.9, 12.8)	
p53								
Wild type	38.7 (20.9, 59.2)	0.748	27.4 (17.4, 35.4)	0.357	19/14	0.486	6.0 (4.0, 9.3)	0.989
Mutant type	48.6 (25.1, 61.6)		24.4 (18.7, 29.2)		8/3		6.2 (3.2, 10.2)	
Ki67								
<5%	43.2 (28.9, 62.2)	0.452	27.4 (20.0, 37.7)	0.740	10/5	0.661	5.9 (3.1, 7.2)	0.685
5–20%	31.3 (22.8. 42.1)		20.6 (17.3, 32.4)		8/4		6.8 (3.0, 10.9)	
>20%	51.0 (16.6, 60.3)		25.4 (19.2. 33.8)		9/8		7.5 (4.0, 10.6)	
MMP-9								
Positive	39.8 (26.3, 61.3)	0.520	25.0 (19.5, 34.5)	0.777	14/10	0.760	5.6 (2.6, 8.8)	0.288
Negative	43.5 (16.3, 59.3)		26.9 (15.7, 34.2)		13/7		8.0 (3.7, 11.0)	

**Table 3:** MVD-CD34 and MVD-CD105 in relation with MRI parameters.

	CD34		CD105	
	r	*p*	r	*p*
Tumor volume (cm^3^)	0.062	0.687	−0.069	0.658
Peritumoral edema (mm)	0.207	0.107	−0.090	0.563
Midline deviation (mm)	0.120	0.440	−0.088	0.568
	%	*p*	%	*p*
Edema Shape (Roundish/Irregular)	3.6/4.1	0.644	4.1/3.1	0.405
Edema Crosses Midline (Present/Absent)	5.3/3.8	0.542	10.7/3.4	0.009

**Table 4:** Correlation coefficients between tumor volume, peritumoral edema and midline deviation.

	Tumor Volume (cm^3^)/Peritumoral Edema (mm)	Tumor Volume (cm^3^)/Midline Deviation (mm)	Peritumoral Edema (mm)/Midline Deviation (mm)
Correlation coefficient (*p*)	CC = −0.06 (*p* = 0.67)	CC = −0.02 (*p* = 0.86)	CC = 0.41 (*p* = 0.005)

## Data Availability

All data produced here are available upon request.

## Abbreviations

MMP-9	Matrix metalloproteinase 9
MRI	Magnetic resonance imaging
IDH1	Izocytrate dehydrogenase 1
ATRX	Alpha thalassemia/mental retardation syndrome X-linked
VEGF	Vascular endothelial growth factor
GBM	Glioblastoma
MGMT	O^6^-methylguanine-DNA methyltransferase
MVD	Microvascular density
MMP-2	Matrix metalloproteinase 2
rCBV	Relative Cerebral Blood Volume
rOEF	Relative Oxygen Extraction Fraction
HIF	Hypoxia Inducible Factor
nCBV	Normal Cerebral Blood Volume
nCBF	Normal Cerebral Blood Flow
ROI	Region of Interest
